# The Behavior of a Zn-Al Anticorrosive Coating in the Wiredrawing Process

**DOI:** 10.3390/ma15186190

**Published:** 2022-09-06

**Authors:** Marius Tintelecan, Dana-Adriana Iluțiu-Varvara, Ioana Monica Sas-Boca, Claudiu Aciu

**Affiliations:** 1 Faculty of Materials and Environmental Engineering, Technical University of Cluj-Napoca, 28 Memorandumului Street, 400114 Cluj-Napoca, Romania or; 2 Faculty of Building Services Engineering, Technical University of Cluj-Napoca, 28 Memorandumului Street, 400114 Cluj-Napoca, Romania; 3 Faculty of Civil Engineering, Technical University of Cluj-Napoca, 28 Memorandumului Street, 400114 Cluj-Napoca, Romania

**Keywords:** steel wiredrawing, Zn-Al alloy, crystallization system, loss by drawing, drawing force, coating

## Abstract

The present paper describes and quantifies the behavior of a Zn-Al anticorrosive coating deposited on the surface of a steel wire before its drawing process. For the complete evaluation of this behavior, the drawing of these samples was performed on four wiredrawing lines, differing by the deformation angle 2 · α of the component dies of each line. For good agreement with industrial practice, the drawing series used a partial reduction of the section of 20%. Two aspects were analyzed: the evolution of the chemical composition and the structure of the removed layer during the drawing of the coated steel wire, and the drawing force necessary to carry out this process. This article helps to elucidate how the Zn-Al anti-corrosion layer responds to the stresses inherent in the process of drawing the steel wire on which it is deposited.

## 1. Introduction

The literature on zinc-coated wiredrawing technology lacks information concerning the surface roughness of zinc-coated wire after the multi-stage drawing process at high drawing speeds (above 5 m/s). Nevertheless, the data from the literature on single-stage drawing at a speed of up to 2 m/s show that the angle and the drawing method influence both the top wire layer and the zinc coating surface roughness [1,2,3,4,5,6].

Zinc, aluminum, and their alloys are frequently used as protection materials due to their relatively good corrosion resistance [2,3,7]. 

At the current stage of research in this field, anticorrosive tests have been performed that clearly define the chemical composition of the Zn-Al alloy; this was the starting point for the present research. It has in its chemical composition 4.8...5% Al, the rest being Zn, and corresponds to the eutectic from the Zn-Al equilibrium diagram. However, it is intended for final anticorrosive protection (namely, of the semi-finished product that no longer undergoes a deformation after deposition) and has special anticorrosive properties. Starting from the initial knowledge related to this alloy, tests were performed to deduce the chemical composition of a new Zn-Al alloy, intended for the specialized anticorrosive protection of a metal semi-finished product that is deformed by wiredrawing. In brief, there was a desire for a new Zn-Al alloy, which confers the combination of the high anticorrosive resistance of the initial alloy with its high deformability after the moment of deposition. The study was based on the fundamental idea that Zn is the majority component in the chemical composition of the Zn-Al alloy; after the thermal deposition of this alloy (by immersing the steel wire in the Zn-Al melt) upon the solidification of the deposited alloy, the crystallization qualities of Zn will predominate. Consequently, it will adopt the crystallization mode of Zn: the hexagonal system. This mode of crystallization is demonstrated by many diffractograms. The mode of deformation by drawing it (considering the mode of crystallization) is the twinning mode. For this reason, in the subsequent drawing process, we demonstrate that we are witnessing a majority scraping of the deposited layer. Ideally, the Zn-Al alloy should adopt the cubic system, and therefore a sliding deformation mode. The idea is to deliberately introduce a higher percentage of Al to stop the hexagonal crystallization of the Zn-Al alloy (owed to the majority of Zn) and, consequently, to eliminate the possibility of deformation by twinning the assembly.

In the present paper, two technical variants were compared and analyzed. These technical variants were variant 1 (which corresponds to a Zn deposition, and is referred to in the literature as Hot Dip) and variant 2 (which corresponds to the deposition of the deduced Zn-Al alloy). Our investigations define this behavior, taking into account the appearance of the layer lost during wiredrawing; we monitor the appearance of the protective layer removed by drawing, and the structural and chemical composition of the lost anticorrosive layer.

## 2. Materials and Methods

Combining the anticorrosive properties of the initial Zn-Al alloy and carefully studying the Zn-Al equilibrium diagram, we performed tests in which we slowly increased the percentage of Al. In total, 5 attempts were required to determine the crystallization mode of the new Zn-Al alloy (demonstrated by diffractometry). Determinations were made with the following percentages of Al: 5.2%, 5.4%, 5.6%, 5.8%, and finally 6.0%. At 6.0% Al content in the Zn-Al alloy, we noticed that the alloy “forgot” the hexagonal crystallization mode (so it “forgot” the deformation mode by twinning) and adopted the cubic crystallization mode (meaning that its deformation was achieved by sliding). In other words: at that moment, we created a new Zn-Al alloy capable of conferring high anticorrosive resistance (typical of the initial Zn-Al alloy) and, simultaneously, adequate drawing behavior. The deduction of the optimal chemical composition of the alloy was made in [8], and the present article demonstrates how beneficially it behaves when drawing it [9,10]. This was achieved by constantly comparing different values of particular quantities to try to define the behavior during drawing of two technical variants that have an identical finality. The practical aim was to produce a metal semi-finished product over which an anticorrosive layer is deposited in a certain shape, and which is subsequently deformed to the final dimensions. The two technical variants compared and analyzed were:

- Technical variant 1 corresponds to a Zn deposition, and is referred to in the literature as the Hot Dip variant. In effect, it involves the deposition of a layer of pure Zn on the surface of a steel wire by immersing it in a Zn melt, at a temperature of about 450 °C. The process runs at a certain speed (thus ensuring a perfectly defined duration of deposition) and a certain degree of subsequent wiping.
- Technical variant 2 corresponds to the deposition of the Zn-Al alloy (containing 6.0% Al). The deposition was made by immersing the steel wire in a melt of the alloy at a temperature of 430 °C; the immersion duration and the deletion method were identical for the two compared variants.

According to the authors, the quantities that define the behavior after the deformation by drawing of the assembly are:

- The appearance of the protective layer removed by drawing;
- The structural and chemical composition of the lost anticorrosive layer;
- The amount of the anticorrosive layer lost by drawing;
- The drawing force required for deformation.

These quantities were monitored, and the clear superiority of the coating with the new Zn-Al alloy (as demonstrated in this article) is the novelty of this research, which reveals the precise deformation of the Zn-Al alloy deposited on the surface of a steel wire in the process of its wiredrawing. Moreover, this article defines the behavior related to the Zn-Al crystallization mode (or, more precisely, in relation to its mode of deformation imposed by the mode of crystallization).

Our investigations define this behavior, taking into account the appearance of the layer lost during wiredrawing, by monitoring the appearance of the protective layer removed by drawing, the structural and chemical composition of the lost anticorrosive layer, the amount of protective layer lost by drawing, and the drawing force. This is achieved by comparing, in each technical stage, the values obtained by applying protection variant 1 (pure Zn protection, Hot Dip) with those obtained by applying protection variant 2 (the Zn-Al alloy).

**Figure 1:**
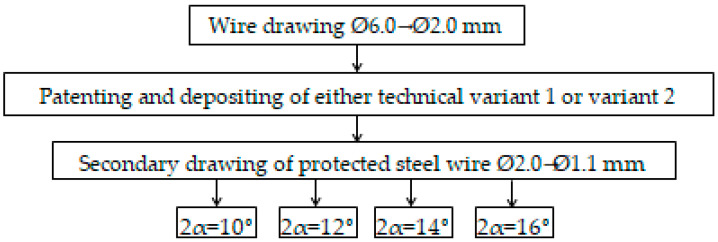
Technical flow with stages of experimentation.

The technical process of comparing the wires (regardless of the coating variant used) involved the following technical flow; the stages of the experiment are presented in Figure 1.

**Figure 2:**
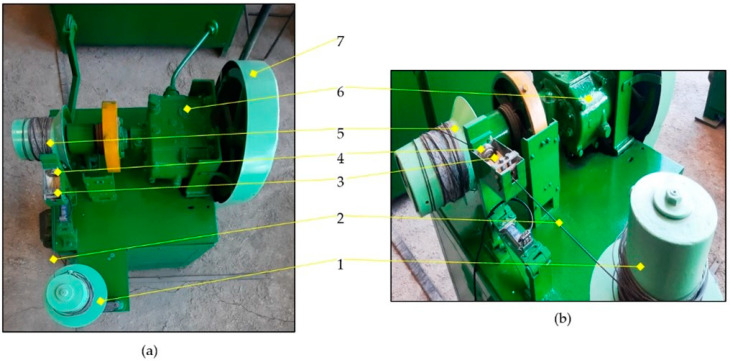
The stand used for wiredrawing: (**a**) top view; (**b**) drawing line detail. Legend: 1—the undriven drum used in the drawing process; 2—the wire sample; 3—the lubricant tank (soap box); 4—the die holder; 5—the driven drum of the drawing process; 6—the gearbox; 7—the pulley–belt system.

The quantification of values was determined using the following parameters:

- The appearance of the protective layer removed by drawing. It has been observed that the appearance of zinc (or, more precisely, iron-zinc compounds) thermally deposited on the surface of a steel wire and lost during its wiredrawing process depends on when it is removed. The volume and color of the removed matter were monitored using photography.
- The structural composition of the lost anticorrosive layer, measured using the electronic microscope JEOL JSM-5600LV (JEOL Ltd., Tokyo, Japan).
- The chemical composition of the lost anticorrosive layer, was deduced using the chemical standard SR EN 10244-2 [11] (gravimetrical procedure), as well as quantitative chemical analyses that determined the percentage of iron present in the removed layer, deposited either in variant 1 or in variant 2.
- The quantity of the lost anticorrosive layer was determined using the analytical balance by Kern & Sohn ADB 100-4 (Kern & Sohn, Ballingen, Germany).
- The drawing force, which was registered using the stand shown in Figure 2.

We note that the value of the drawing force was registered for drawing samples with a diameter of 2.0 mm and a length of 1000 mm, which were made of C50 steel (according to EN 10 083-2). The samples were drawn through a complete series of dies, with four distinct values of deformation angle: 2 · α = 10°, 2 · α = 12°, 2 · α = 14°, or 2 · α = 16°. The diameters were Ø1.8 mm, Ø1.6 mm, Ø1.42 mm, Ø1.26 mm, and Ø1.1 mm. For all of the wiredrawings, the same lubricant was used.

**Figure 3:**
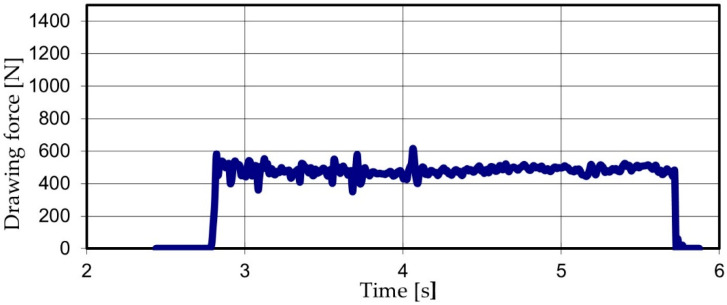
The drawing force recorded for a sample, drawn through a die with the deformation angle 2 · α = 10°.

Thus, we produced 120 graphs of the type shown in Figure 3, representing the value of the drawing force recorded over the entire length (1000 mm) of the sample.

## 3. Results and Discussion

### 3.1. The Appearance of the Protective Layer Removed by Drawing

#### 3.1.1. For Technical Variant 1 (Using Pure Zn)

**Figure 4:**
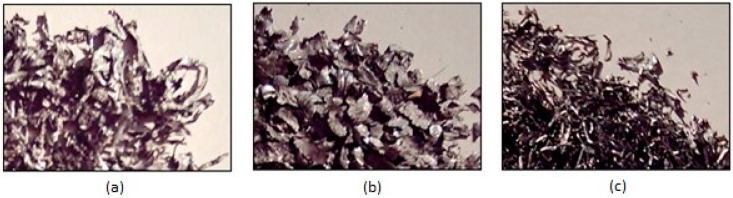
Ways that zinc was lost, obtained after steel wiredrawing: (**a**) granules; (**b**) crusts; (**c**) continued splinters.

It has been observed that, at the first drawing stages, the protective layer was removed in the form of granules (Figure 4a) and crusts (Figure 4b). 

In the last stages of the drawing series, the removal of the protective layer in the form of continued splintering (Figure 4c) was observed. These splinters were in fact present throughout the series of wiredrawing when, in the path traveled through the die (regardless of the number of passes), the steel wire protected in this technical variant encountered a sharp, hard body, or when the die had a hard particle with sharp corners (for example, a steel chip or iron oxide) glued to its inner surface (in the deformation zone). 

The resulting granules and crusts contain a mixture of zinc and wiredrawing lubricant and have a dirty white color due to the partial combustion of the lubricant and zinc oxidation, produced by the high temperature in the deformation center. At the time of release, the zinc splinters have a bright white color specific to a strongly hardened surface; over time, they become matte due to oxidation.

#### 3.1.2. Technical Variant 2 (Using the Zn-Al Alloy)

When using technical version 2 for corrosion protection, the deposited Zn-Al alloy is removed from the surface of the steel wire at the time of its drawing in a much smaller amount; after drawing, it is only found in the lubricant volume in the form of small granules.

### 3.2. The Structural Composition of the Lost Anticorrosive Layer

#### 3.2.1. For Technical Variant 1 (Using Pure Zn)

For the Hot Dip deposition (technical variant 1), the Zn layer on the surface of the steel wire is a strongly (structurally) heterogeneous assembly. In practical terms, in its cross section, we can see the existence of seven concentric substrates that differ by chemical composition, network structure, phase micro hardness, and their electrochemical potential, which have the symbols (from the iron-zinc interface to the outside) α, γ, Γ, δ_1_, δ, ξ and η [12,13].

**Figure 5:**
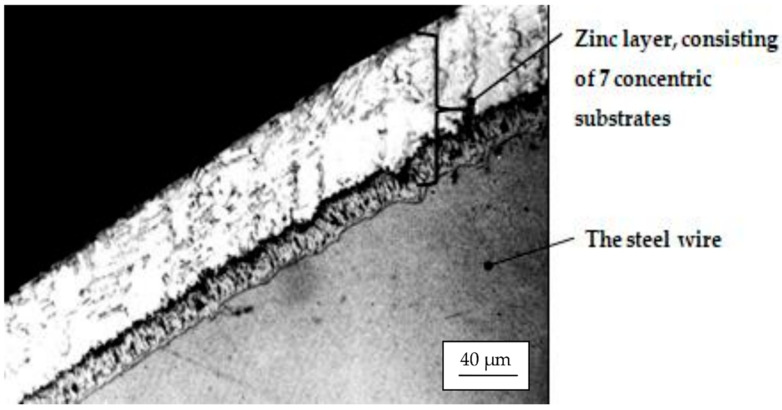
The structure of the Zn layer, “Hot Dip”, deposited on the steel wire surface.

**Figure 6:**
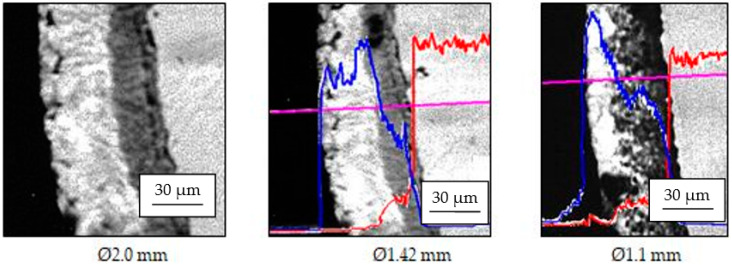
The structure of the Zn layer (deposited on variant 1) on the steel wire surface; the compositional profile of Zn (blue) and Fe (red), along its drawing on analysis line (purple).

It is logical that, in this case, drawing will remove the outer layers that are most exposed to contact with the die. These are the η layer (the outer layer which has a maximum composition of 0.003% Fe, the rest being Zn, a hexagonal crystalline structure, and a microhardness of 37–40 HV units) and the ξ layer (which has a maximum chemical composition of 6.0–6.2% Fe, the rest being Zn, a crystal lattice with a hexagonal structure, and a microdurity of 200, ..., 427 HV units) (Figure 5). This assumption was confirmed by analyzing the microphotographs obtained from the electron microprobe regarding the appearance of the zinc layer (in cross section) and the compositional profile of the wires obtained from it (compositional profiles of Zn and Fe) that had a diameter smaller than the initial Ø1.42 mm: Ø1.1 mm (Figure 6).

#### 3.2.2. Technical Variant 2 (Using the Zn-Al Alloy)

**Figure 7:**
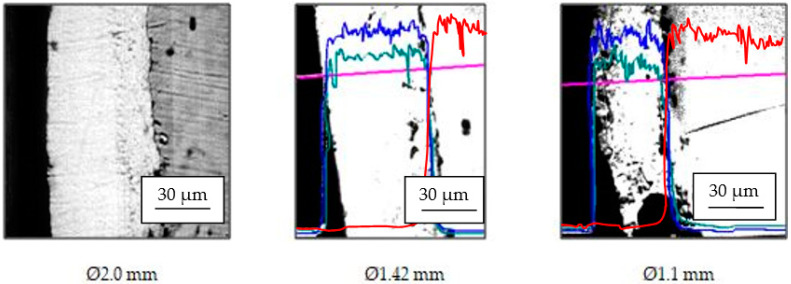
The structure of the anticorrosive layer (deposited on variant 2) on the steel wire surface; the compositional profiles of Zn (blue), Al (green), and Fe (red) along its drawing on analysis line (purple).

Immediately noticeable in the deposition of technical variant 2 is the high structural homogenity, with practically the whole coating layer having a unique, uniform structure, which is also visible in the exposed microphotographs (Figure 7).

### 3.3. The Chemical Composition of the Anticorrosive Layer Lost by Drawing

We consider the percentage of iron to be relevant to the evolution of the chemical composition of the protective layer removed by drawing, as found in the granules/crusts/splinters of the removed layer, and in the lubricant located after each die (practically after each pass) [14,15].

**Figure 8:**
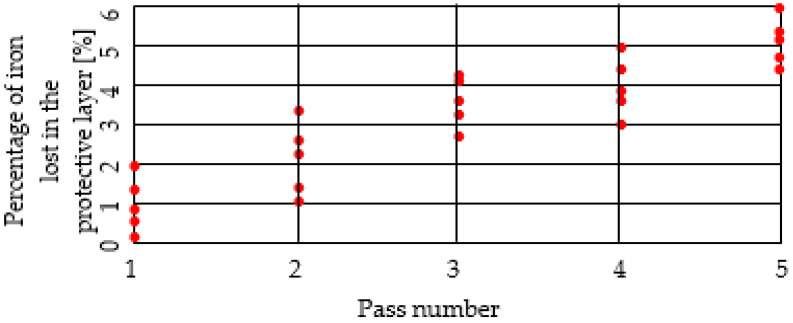
The evolution of Fe percentage lost in the steel wiredrawing process (variant 1 of the protective layer).

**Figure 9:**
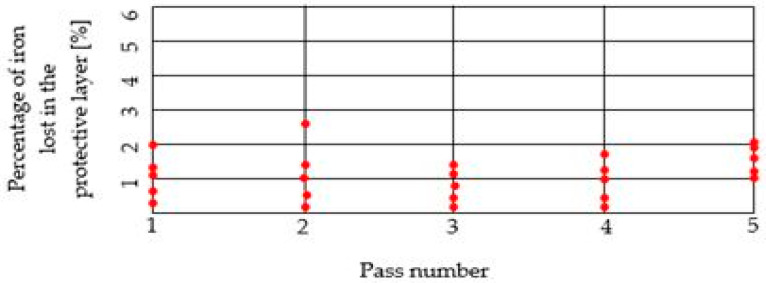
The evolution of Fe percentage lost in the steel wiredrawing process (variant 2 of the protective layer).

We made five such determinations, which are shown in Figure 8 and Figure 9.

#### 3.3.1. For Technical Variant 1 (Using Pure Zn)

The percentage of iron increases with each pass, demonstrating the removal of the surface layers of pure Zn [16,17]. The resulting wire becomes matte and less resistant to corrosion (Figure 8).

#### 3.3.2. For Technical Variant 2 (Using Zn-Al Alloy)

The percentage of iron increases with each pass; the increase is relatively constant, demonstrating the relative chemical uniformity of the Zn-Al alloy layers removed by drawing [18,19,20,21].

In Table 1 are presented the values of standard deviation for each pass, and for each protection variant (Variant 1 of the protective layer, data from Figure 8; Variant 2 of the protective layer, data from Figure 9).

### 3.4. The Chemical Composition of the Anticorrosive Layer Lost by Drawing

The quantity of the anticorrosive layer lost by drawing was determined gravimetrically, according to SR EN 10244-2 [11]. Table 2 shows the percentage values of loss by the drawing of the protective layer for deposition variants 1 and 2. For variant 1 of the protective layer, values were determined statistically (regardless of the deformation angle of the dies). For variant 2 of the protective layer, values were determined as an average of five measured values.

The standard deviation for the first series (for variant 1) is 4.16; meanwhile, for the second series (for variant 2), it is 1.72.

**Figure 10:**
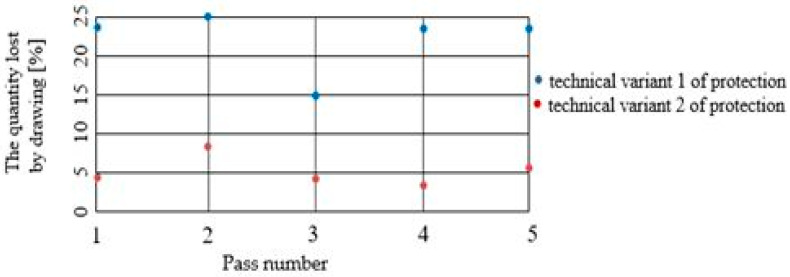
The evolution of the quantity of the protector layer lost in the steel wiredrawing process.

The statistically determined average numerical values can be represented in graphs, such as that shown in Figure 10.

It can be seen that the field of the percentage of the anticorrosive layer lost by drawing is 15…25% in the case of protection variant 1, and about 3…8% in the case of protection variant 2.

### 3.5. The Evolution of the Drawing Force for the Wires Protected with Technical Variant 1, Presented Comparatively with the Wires Coated with Technical Variant 2

**Figure 11:**
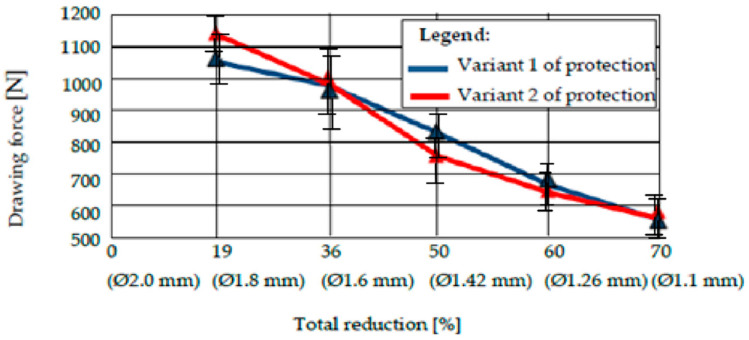
The drawing force of protection variants 1 and 2 for a deformation angle 2 · α = 10°.

**Figure 12:**
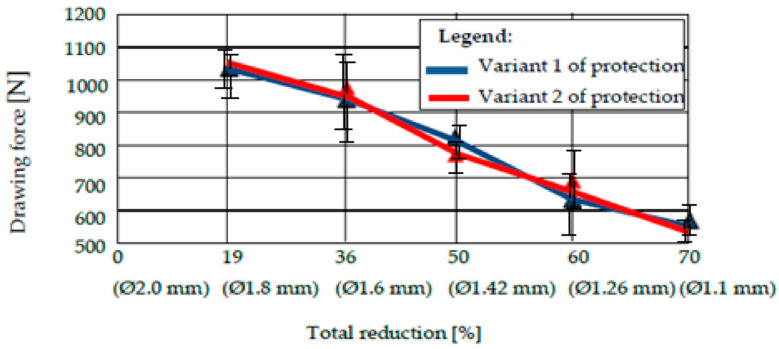
The drawing force of protection variants 1 and 2 for a deformation angle 2 · α = 12°.

**Figure 13:**
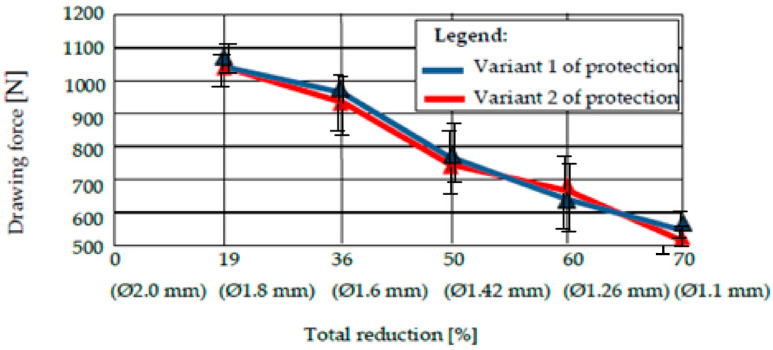
The drawing force of protection variants 1 and 2 for a deformation angle 2 · α = 14°.

**Figure 14:**
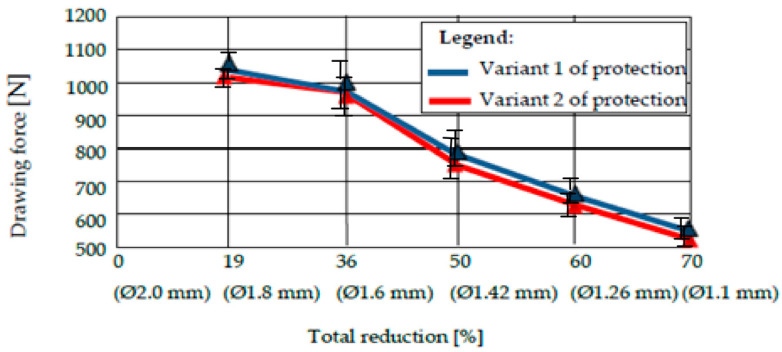
The drawing force of protection variants 1 and 2 for a deformation angle 2 · α = 16°.

We performed an arithmetic mean of three values of the drawing force recorded for drawing a sample covered with a certain type of deposition with a certain diameter, and a die with a certain deformation angle [22]. The results are shown in Figure 11, Figure 12, Figure 13 and Figure 14.

The analysis of the values of the drawing force presented in the tables reveals two conclusions:

1. The drawing forces have equal values for both protection variants, regardless of the angle of the deformation zone. However, there is an optimum for protection variant 1 (with pure zinc), i.e., a minimum value of the required drawing force for 2 · α = 12°.
2. Comparing very precisely the values of the drawing forces, recorded for different values of the deformation angle, the authors observed a minimization of the drawing force for the protection variant 1 for 2 · α = 12°, and for the protection variant 2 with the deformation angle 2 · α = 14°, when the same lubricant was used for both variants.

## 4. Conclusions

We undertook a process of comparing two technical variants of a protective layer for steel wire (variant 1 = “Hot Dip” deposition of pure Zn; variant 2 = deposition of Zn alloy: 94% + Al: 6%). The wires were subsequently drawn, in order to determine the effect of the drawing process on the loss of the protective layer, as well as the structural and chemical composition of the lost layer, and the amount of the layer lost during drawing. We also examined the necessary drawing force. It was observed that:

1. From a structural point of view, we noted the existence of an accentuated non-uniformity in the protective layer deposited in variant 1; SEM analysis reveals the existence of this non-uniformity in the entire volume deposited in this variant of the Fe-Zn alloy, in which the percentage of Fe increases towards the interface of the protective layer of the steel wire. For protection variant 2, the SEM analysis reveals an accentuated structural uniformity.
2. By comparatively analyzing the two protection variants, the superior chemical uniformity of the protective layer from protection variant 2 was clearly demonstrated. The percentage of iron in the protective layer lost by drawing evolves up to 6% for protection variant 1, and remains constant for protection variant 2.
3. The range of the percentage of the corrosion layer lost by drawing is 15…25% in the case of protection variant 1, and approximately 3…8% in the case of protection variant 2.
4. The forces required to draw the protected steel wires with one of the above variants (variant 1: Zn; variant 2: Zn-Al alloy) are quite similar, and no clear distinction can be made when comparing the two protection variants. In terms of the forces required to draw the steel wires protected with one of the analyzed technical variants, it was found that they were minimized for the deformation angle of the 2 · α dies: 14° for variant 1, and 12° for protection variant 2.

## Figures and Tables

**Table 1:** Values of standard deviation.

Number of Passes	Variant 1 (Figure 8)	Variant 2 (Figure 9)
1	0.67	0.66
2	0.93	0.90
3	0.60	0.59
4	0.72	0.71
5	0.63	0.43

**Table 2:** Percentage values of the loss by drawing of the protective layer.

The Steel Wire Diameter [mm]	Number of Passes	The Quantity Lost by Drawing [%] Variant 1	The Quantity Lost by Drawing [%] Variant 2
2.0	-	-	-
1.8	1	24	4.5
1.6	2	25	7.8
1.42	3	15	4.3
1.26	4	24	3.2
1.1	5	24	5.2

## Data Availability

The data presented in this study are available on request from the corresponding authors.
